# Canopy Apparent Photosynthetic Characteristics and Yield of Two Spike-Type Wheat Cultivars in Response to Row Spacing under High Plant Density

**DOI:** 10.1371/journal.pone.0148582

**Published:** 2016-02-04

**Authors:** Tiening Liu, Zhenlin Wang, Tie Cai

**Affiliations:** 1 Key Laboratory of Crop Physi-ecology and Tillage Science in Northwestern Loess Plateau, Ministry of Agriculture/College of Agronomy, Northwest A&F University, Yangling, 712100, Shaanxi, China; 2 The Chinese Institute of Water-saving Agriculture, Northwest A&F University, Yangling, 712100, Shaanxi, China; 3 State Key Laboratory of Crop Biology, Agronomy College of Shandong Agricultural University, Taian, 271018, Shandong, China; University of California - Davis, UNITED STATES

## Abstract

In northern China, large-spike wheat (*Triticum aestivum* L) is considered to have significant potential for increasing yields due to its greater single-plant productivity despite its lower percentage of effective tillers, and increasing the plant density is an effective means of achieving a higher grain yield. However, with increases in plant density, the amount of solar radiation intercepted by lower strata leaves is decreased and the rate of leaf senescence is accelerated. Row spacing can be manipulated to optimize the plant spatial distribution under high plant density, therefore improving light conditions within the canopy. Consequently, field experiments were conducted from 2010 to 2012 to investigate whether changes in row spacing under high plant density led to differences in canopy apparent photosynthesis (CAP), individual leaf photosynthesis and grain yield. Two different spike-type winter wheat cultivars, Jimai22 (a small-spike cultivar as a control cultivar) and Wennong6 (a large-spike cultivar), were grown at a constant plant density of 3,600,000 plants ha^–1^ (a relatively higher plant density) over a wide range of row spacing as follows: 5-cm row spacing (R_0_), 15-cm row spacing (R_1_), 25-cm conventional row spacing (R_2_), and 35-cm row spacing (R_3_). The two-year investigations revealed that increased row spacing exhibited a significantly higher light transmission ratio (LT), which improved light conditions within the canopy; however, excessive light leakage losses in R_2_ and R_3_ treatments were not favorable to improved irradiation energy utilization efficiency. Aboveground biomass accumulation was influenced by row spacing. Two spike-type wheat accumulated greater biomass under 15-cm row spacing compared to other row spacing treatments, although a markedly improved photosynthetic rate (*P*_N_), effective quantum yield of photosystem II (Φ_PSII_) and maximal efficiency of photosystem II photochemistry (F_v_/F_m_) in the penultimate and third leaves were observed in R_2_ and R_3_ treatments. At the same time, a longer duration of CAP and green leaf area was maintained in R_1_ during grain filling. Compared with conventional row spacing, Wennong6 in R_1_ treatment obtained 21.0% and 19.1% higher grain yield in 2011 and 2012, respectively, while for Jimai22 it increased by 11.3% and 11.4%, respectively. A close association of yield with CAP and LAI at mid-grain filling was observed. In conclusion, for the tested growing conditions, decreasing the row spacing to an optimal distance (15 cm) maintained a longer duration of LAI and CAP during grain filling, made a better coordination of group and individual leaf photosynthesis, and accumulated higher aboveground biomass, leading to a greater grain yield. In addition, Wennong6 had a more rational canopy architecture than Jimai22 (improved LT and higher LAI) and CAP under 15-cm row spacing, leading to a higher grain yield, which indicated that the large-spike type cultivar has the potential to obtain higher yields by increasing plant density through optimum row spacing allocation (15 cm).

## Introduction

Large and small-spike type winter wheat cultivars (*Triticum aestivum* L.) are widely planted in theNorth China Plain. The number of spikes per hectare of small-spike wheat is almost at its maximum [1]; consequently, it has been proposed that large-spike wheat has the potential to increase yields due to its better single-plant productivity despite having a lower effective tiller percentage, and that this could be achieved by increasing plant density [2–3]. However, the amount of solar radiation intercepted by lower strata leaves decreases during the day, and this accelerates leaf senescence when the number of plants is increased [4–5].

Under high plant density, an adequate leaf area index and an effective blade spatial arrangement are conducive to shaping an appropriate canopy architecture, which is strongly associated with the amount of radiation intercepted [6–7]. Row spacing can be manipulated to optimize plant spatial distribution and improve light conditions within the canopy. In production agriculture, 25-cm row spacing is the conventional planting pattern for wheat among smallholders in the North China Plain [8]. Some previous studies have demonstrated that wide row spacing improved light conditions within a fully developed wheat canopy [9–10], which was also observed by Reta-Sanchez and Fowler [11] for cotton, and Awalet *et al*. [12] and Liu and Song [5] for maize. However, other researchers found that radiation interception and yield of cereal crops increased with reduced row spacing [13–15], accompanied by a reduction in the solar energy utilization efficiency and increased respiration within the canopy [16–17]. Moreover, no yield increase of winter wheat (*Triticum aestivum* L.) at 10-cm row spacing was found compared with 30-cm row spacing under conventional tillage systems [18], and McLeod *et al*. [19] found that row spacing had little effect on grain yield of winter wheat. Moreover, other studies have examined the effect of row spacing on leaf senescence and dry matter accumulation [20–21]. The creation of rational canopy architecture through optimum row spacing allocations under high plant density to enable better coordination between population and individual plant growth has become an important issue.

Consequently, two different spike-type winter wheat cultivars, Jimai22 (a small-spike cultivar as a control cultivar) and Wennong6 (a large-spike cultivar), were grown at a constant plant density of 3,600,000 plants ha^–1^ (a relatively higher plant density) over a wide range of row spacing as follows: 5-cm row spacing (R_0_), 15-cm row spacing (R_1_), 25-cm conventional row spacing (R_2_), and 35-cm row spacing (R_3_). The objectives of our study were: 1) to analyze the responses of CAP, photosynthetic performances of the top three leaves and the yield of the two cultivars to row spacing under high plant density; 2) to determine which row spacing enabled better coordination between CAP and photosynthetic characteristics of the top three leaves under high plant density and achieved greater biomass accumulation and grain yield, and 3) to identify whether large-spike type wheat has larger yield potential when it was planted underr high density through optimum row spacing. Our results will provide a theoretical basis for super-high-yield cultivation with different spike types of wheat.

## Materials and Methods

### Experimental site

**Fig 1:**
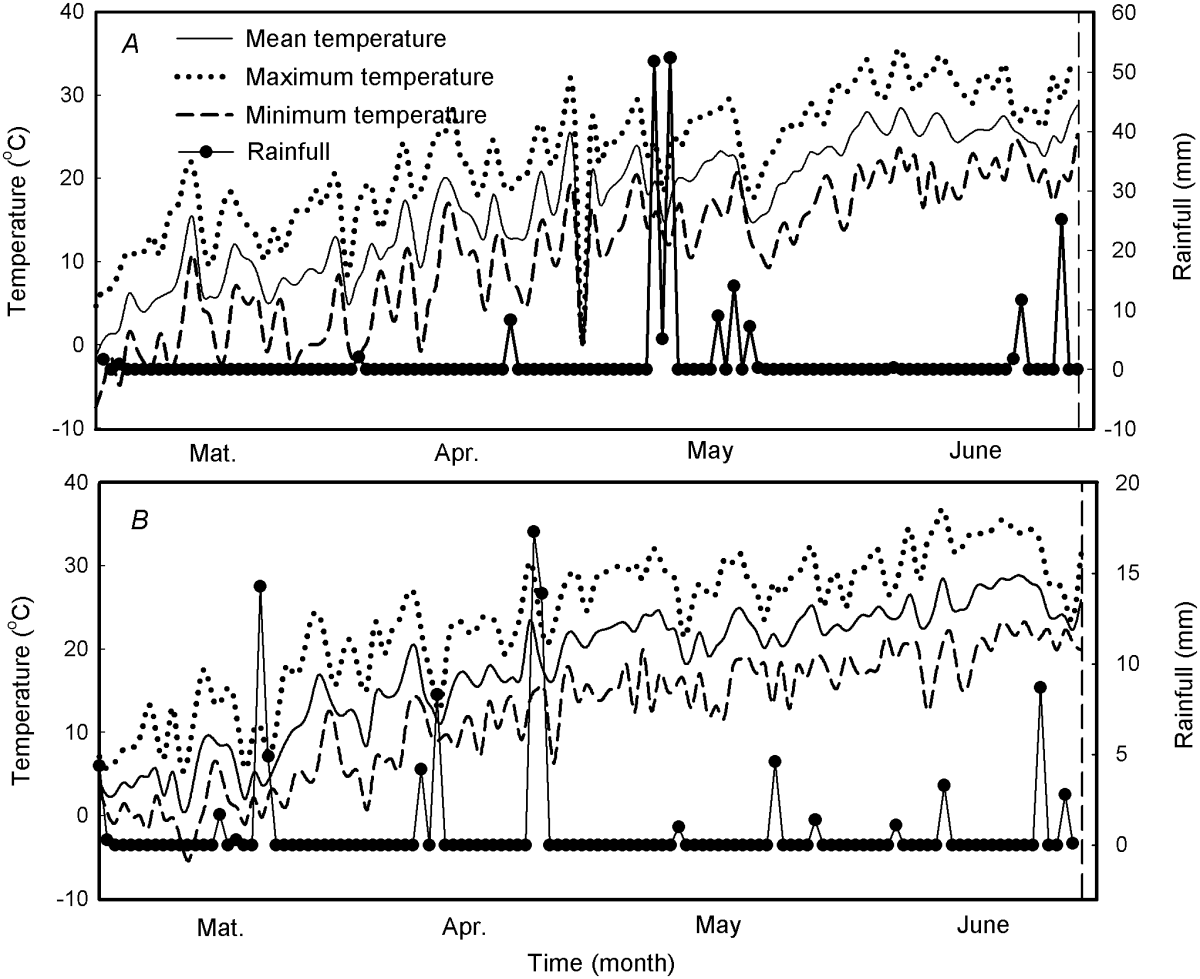
Rainfall, maximum, minimum and mean temperatures (°C) recorded during the growing season (March to June) in 2010–2011 (*A*) and 2011–2012 (*B*).

A 2-year field experiment was conducted at Tai’an Experimental Station of Shandong Agricultural University, Tai’an, China (36°18′N, 117°13′E, 128 m above sea level) on a soil of sandy loam during the 2010–2011 and 2011–2012 growing seasons. The soil surface (0–20 cm) had an organic matter content of 12.54 g kg^–1^, 0.87 g kg^–1^ total nitrogen, 76.1 mg kg^-1^ available nitrogen, 25.6 mg kg^–1^ phosphate and 97.2 mg kg^–1^ potassium in both years. Basal fertilization included N as urea, phosphorus as calcium superphosphate and potassium as potassium chloride at a coverage of 112.5 kg N ha^–1^, 105 kg P_2_O_5_ ha^–1^ and 150 kg K_2_O ha^–1^, respectively. In addition, another 112.5 kg N ha^–1^ as urea was applied at the jointing stage on 6 April 2011 and 7 April 2012. The rainfall (mm) and air temperature (°C) during the growing season were measured by an automatic weather station (Fig 1).

### Ethics Statement

The experimental field used in this study belongs to the Shandong Agricultural University, which is a comprehensive research institution, and it has a research ethics review committee to ensure experiments do no harm to crops, animals and humans. Our study was approved by this university, so no specific permissions were required for the described field experiments. The sampling locations were not privately-owned or protected in any way, and this field study did not involve any endangered or protected species. In addition, there was also no vertebrate species in this study.

### Experimental design

**Fig 2:**
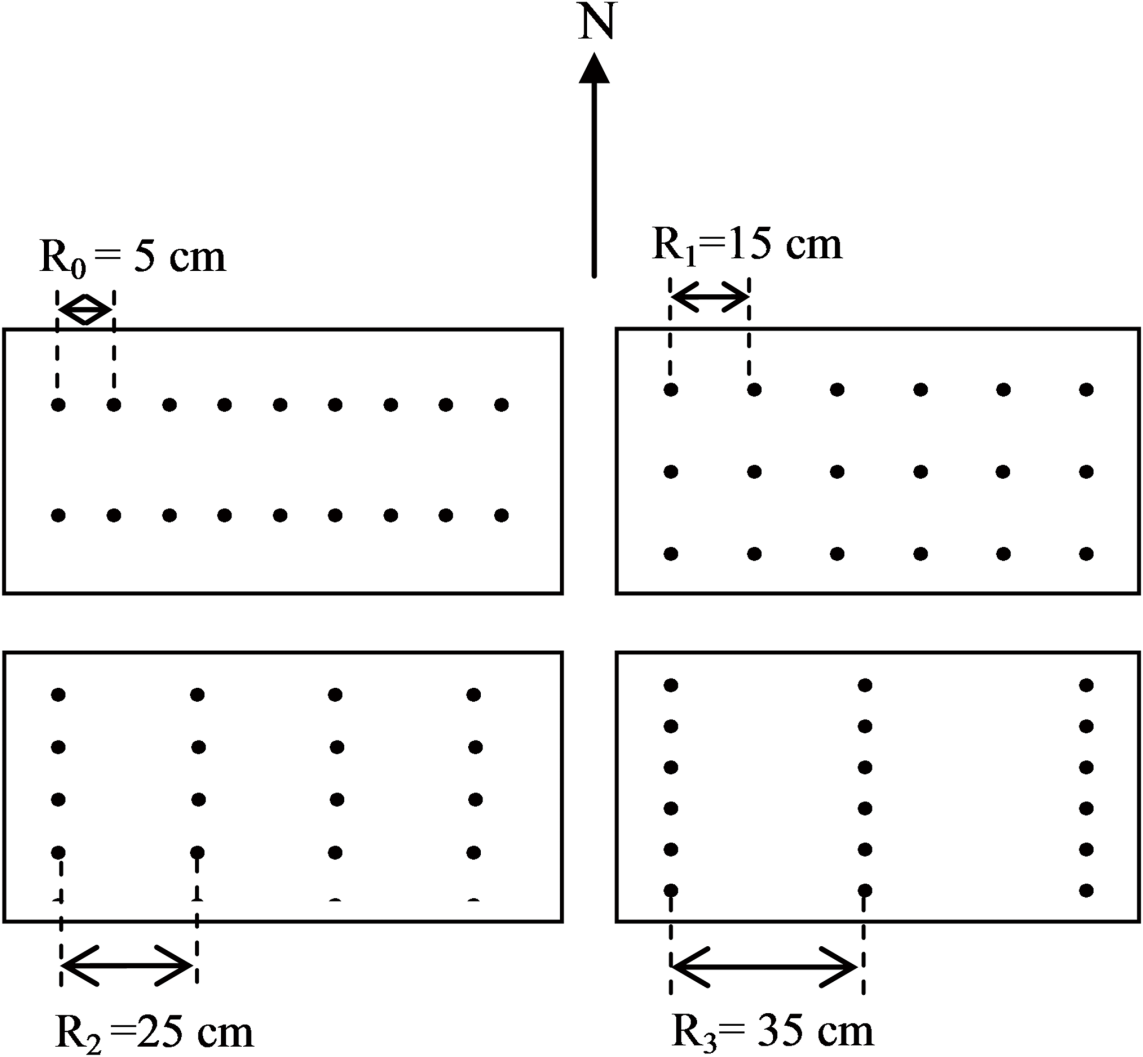
A schematic diagram showing R_0_ (5-cm row spacing), R_1_ (15-cm row spacing), R_2_ (25-cm row spacing) and R_3_ (35-cm row spacing) planting patterns at a density of 3,600,000 plants ha^-1^ over two years.

Two widely planted cultivars in local production, Jimai22 (a small-spike cultivar as control cultivar) and Wennong6 (a large-spike cultivar), were grown at a density of 3,600,000 plants ha^–1^ in this experiment. Four row spacing configurations (Fig 2) were designed as a randomized complete block design with three replications: 5-cm row spacing (R_0_), 15-cm row spacing (R_1_), 25-cm traditional row spacing (R_2_) and 35-cm row spacing (R_3_). The experimental units were 5-m long and 5-m wide. The two cultivars were sown on 9 October 2010 and 10 October 2011; the date of harvest was 10 June 2011 and 12 June 2012, respectively. Irrigation was conducted three times before winter, at the jointing and booting stage during the two growing seasons. The disease, pests and weeds controls in each treatment were well controlled by managers.

### Measurements

#### Canopy apparent photosynthesis (CAP)

Canopy apparent photosynthesis was measured in a modified closed gas exchange system using an infrared gas analyzer (GXH-305; Beijing Analytical Instrument Co., Beijing, China) with the following modifications [22]. The aluminium framed chamber was 1.2 × 1 m in area and 1.2 m in height. The chamber was covered with 0.6 mm of Mylar, which permitted sunlight into the chamber at up to 95% of its natural intensity. Two battery-powered 60-W fans maintained the airflow inside the chamber. Decreases in CO_2_ concentration were linear and were usually measured within 1 min after closing the chamber. Measurements were conducted between 9:30 am and 11:30 am with three replicates in each treatment at 10-day intervals from 10 days before anthesis to 30 days after anthesis (DAA). CO_2_ exchange rates were expressed on a soil area basis. The canopy apparent photosynthesis was calculated as follows:

CAP=slope×n/A,

where slope is the decrease in CO_2_ concentration per unit time (μmol mol^-1^ s^-1^), n is the number of moles of air in the chamber, and A is the ground area. The variable n is calculated as PV / RT, where P is pressure in k Pa, V is volume of the chamber in L, T is the Kelvin temperature in the chamber in K, and R is the gas constant (8.314 k Pa L mol^-1^ K^-1^).

#### Net photosynthetic rate (PN) in flag leaf, penultimate leaf and third leaf

The net photosynthetic rate of flag leaf, penultimate leaf and third leaf from three healthy and uniform plants in each plot was measured using a portable photosynthesis system (CIRAS-II; PP Systems, Hitchin, UK). The chamber was equipped with a red/blue LED light source. The PAR was set at 1,200 μmol m^–2^ s^–1^. Measurements were taken between 9:30 am and 11:30 am on sunny days at 10-day intervals from anthesis to 30 DAA.

#### Chlorophyll (Chl) fluorescence parameters in flag leaf, penultimate leaf and third leaf

The same leaves as for *P*_N_ measurement were taken for chlorophyll fluorescence parameters using a portable pulse-modulated fluorometer (FMS-2, Hansatech, Norfolk, UK). The maximum quantum efficiency (F_v_/F_m_) was calculated according to Krause and Weis [23]. The effective quantum yield of photosystem II photochemistry (Φ_PSII_) was calculated as defined by Genty *et al*. [24]. The minimum and maximum fluorescence (F_0_ and F_m_) were determined after full-dark adaptation for 15 min, and the steady-state fluorescence (F_s_) and the maximum fluorescence in a light-adapted state (F_m_') were determined under actinic light of 1,200 μmol m^-2^ s^-1^ for 10 min and a 1-s pulse of saturating radiation of 4,000 μmol m^-2^ s^-1^, respectively.

#### Leaf area index (LAI)

Five representative single stems from the central rows of each plot (a total of 15 single stems in each treatment) were selected for measurement of leaf area from anthesis to maturity at 10-day intervals. Leaf length (L) and maximum width (W) were recorded and used to calculate LA; i.e., *LA* = *L* × *W* × 0.83. The equation for LAI was as follows:

LAI=GLA×N/S,

where N is the number of plants within a unit area of land and S is the unit area of land.

#### Light transmission ratio (LT)

The LT at different canopy layers was estimated from incident photosynthetic active radiation (IPAR) and total photosynthetic active radiation (TPAR) at a mid-filling stage. IPAR was measured in the flag leaf (F-) layer, penultimate leaf (P-) layer, third leaf (T-) layer, and bottom leaf (B-) layer (5 cm from the ground). In addition, TPAR was estimated at the top of the canopy. Three independent determinations within each plot were made with a line quantum sensor (LP-80; Decagon Devices, Inc., Pullman, WA, USA) between 9:30 am and 11:30 am on sunny days. These measurements were made diagonally across the rows. The light transmission ratio was calculated using the following equation:

LT=IPAR/TPAR×100%.

#### Grain yield, yield components and aboveground biomass

At physiological maturity, the number of spikes in each plot within an experimental unit (5-m long and 5-m wide region) were counted and converted to spike density (spikes ha^–1^). Thirty stems from the central rows of each experimental unit were then randomly selected and each stem was threshed separately by hand. The grain weight and rest of each stem (except root) were oven-dried at 75°C for 72 h and recorded. Finally, the average 1,000-kernel weight of the 30 stems in each plot was calculated, and the dry matter was determined by weighting. The aboveground dry matter was then converted to biomass yield in kg ha^-1^. After the determination of yield components, all other ears in each experimental unit were threshed and oven-dried. The grains in each plot were then combined with the grains from the 30 stems used for yield components determination to calculate the total grain yield in Kg ha^-1^.

### Statistical analysis

The mean and standard errors were calculated for individual measurements on each sampling date. The analysis of variance was performed with SPSS 17.0 (SPSS Institute Inc.). To determine significant treatments effects, multiple comparisons among the treatments were performed with the least significant difference (LSD) test, and the significance level was set at the 0.05 probability level.

## Results

### Canopy apparent photosynthesis (CAP)

**Fig 3:**
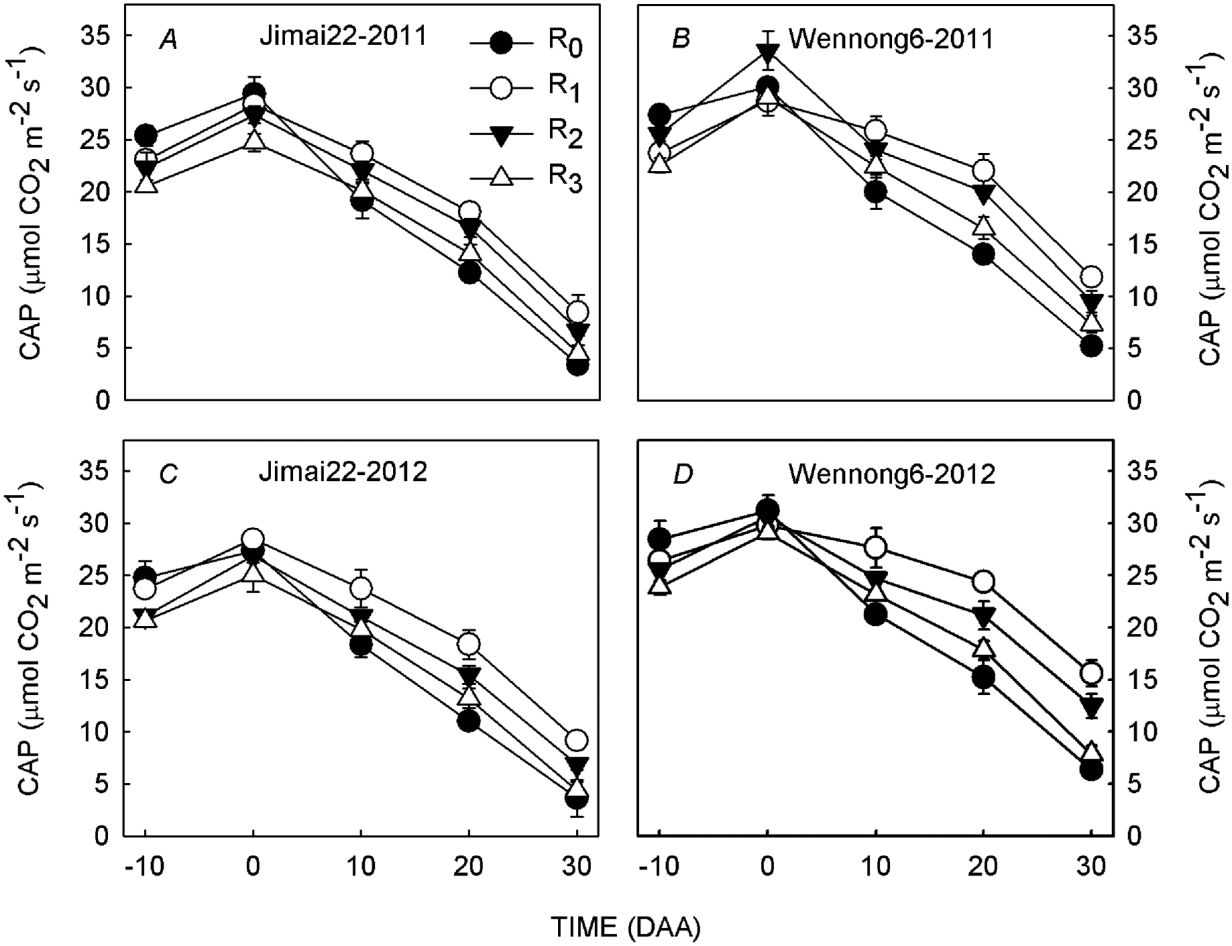
Effects of row spacing on canopy apparent photosynthesis (CAP) of two spike types of wheat under high plant density over a two-year period (2011 and 2012). R_0_, R_1_, R_2_ and R_3_ refer to 5-cm row spacing, 15-cm row spacing, 25-cm row spacing and 35-cm row spacing, respectively. The data are means ± standard error (n = 3). Vertical bars indicate the SE.

Row spacing configurations had significant effects on CAP during 2011 and 2012 growing periods. The highest CAP was observed at anthesis, and then it declined to different degrees. The 5-cm row spacing (R_0_) obtained higher CAP only at 10 days after anthesis (DAA) and before anthesis, and then it produced a drastic decline. Compared with 25-cm conventional row spacing (R_2_), 15-cm row spacing (R_1_) maintained longer duration of CAP, while that in 35-cm row spacing (R_3_) was significantly lower, indicating that decreasing row spacing to an optimum distance under high plant density was beneficial for the improvement of group photosynthetic capacity during grain filling (Fig 3). In addition, the magnitude of improvement, varied among variables. In contrast to Jimai22, a longer duration of CAP in Wennong6 under R_1_ was observed.

### Net photosynthetic rate (*P*_N_) of the top three leaves

**Fig 4:**
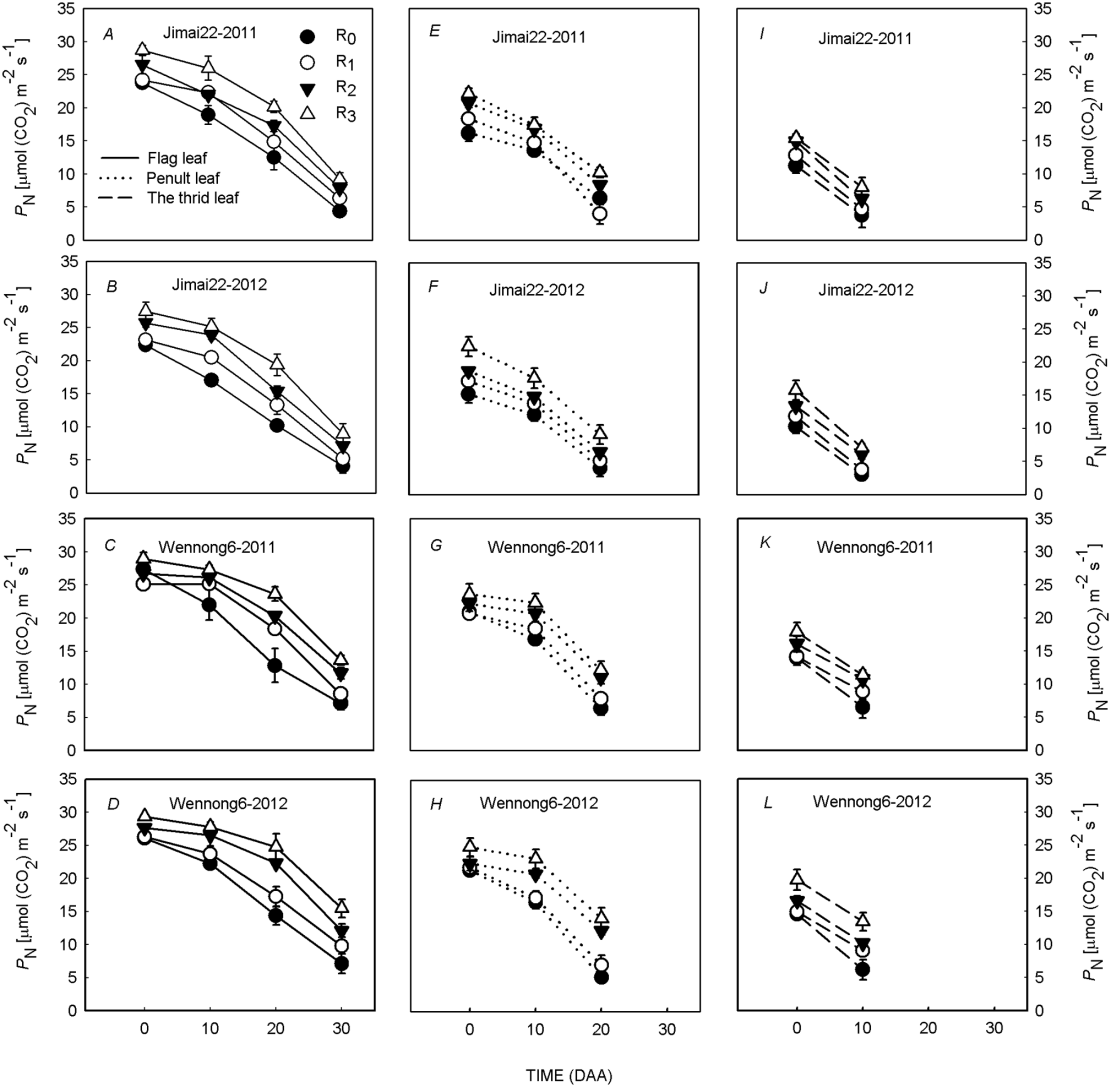
Effects of row spacing on photosynthetic rate (*P*_N_) of flag (*A*,*B*,*C*,*D*), penultimate (*E*,*F*,*G*,*H*) and third leaves (*I*,*J*,*K*,*L*) of two spike types of wheat under high planting density over a two-year period (2011 and 2012). R_0_, R_1_, R_2_ and R_3_ refer to 5-cm row spacing, 15-cm row spacing, 25-cm row spacing and 35-cm row spacing, respectively. Data are means ± standard error (SE; n = 3). Vertical bars indicate the SE.

The same *P*_N_ tendency was found under different treatments in the two cultivars (Fig 4). In both cultivars, the *P*_N_ in the flag leaf was higher than that in penultimate leaf and third leaves throughout the grain filling period. Moreover, the *P*_N_ of the top three leaves under different treatments displayed the following trend: R_3_>R_2_>R_1_>R_0_, indicating that the *P*_N_ of the penultimate and third leaves was enhanced in wide row spacing under high plant density.

### Chlorophyll fluorescence parameters of the top three leaves

**Fig 5:**
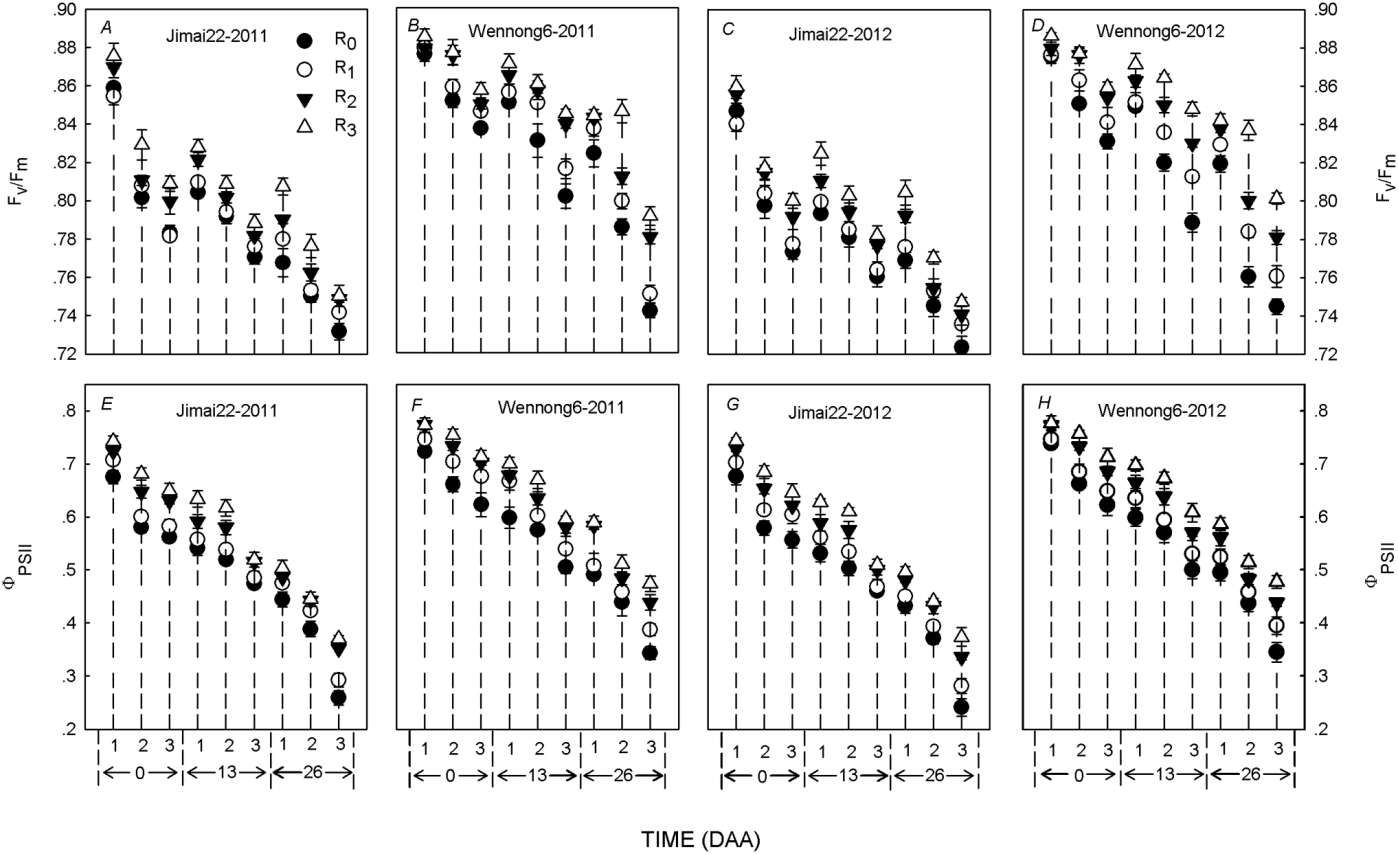
Effects of row spacing on *F*_v_/*F*_m_ (*A*,*B*,*C*,*D*) and *Φ*_PSII_ (*E*,*F*,*G*,*H*)of the flag, penultimate and third leaves of two spike types of wheat under high plant density over a two-year period (2011 and 2012). R_0_, R_1_, R_2_ and R_3_ refer to 5-cm row spacing, 15-cm row spacing, 25-cm row spacing and 35-cm row spacing, respectively. Numbers 1, 2 and 3 refer to the flag, penultimate and third leaves, respectively. The data are means ± standard error (SE; n = 3). Vertical bars indicate SE.

The F_v_/F_m_ and Φ_PSII_ after anthesis were regulated by row spacing, but the magnitude of this regulation varied markedly among treatments (Fig 5). The F_v_/F_m_ and Φ_PSII_ responded similarly to row spacing in both cultivars. The F_v_/F_m_ and Φ_PSII_ in R_3_ treatment for the top three leaves was significantly higher than in other treatments, while in different layers of the canopy the following trend was observed: flag leaf >penultimate leaf >third leaf.

### Leaf area index (LAI)

**Fig 6:**
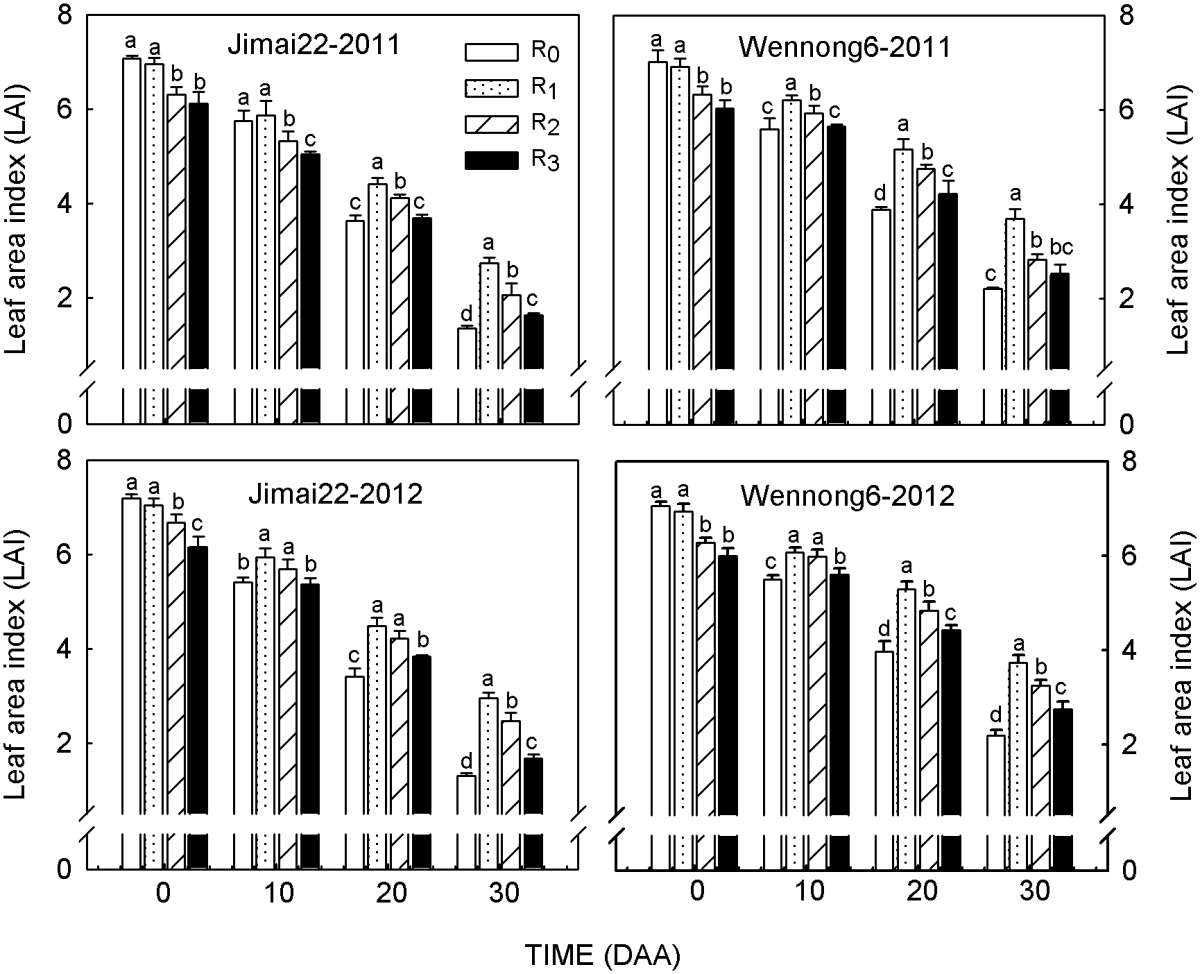
Effects of row spacing on leaf area index (LAI) of two spike types of wheat under high plant density over a two-year period (2011 and 2012). R_0_, R_1_, R_2_ and R_3_ refer to 5-cm row spacing, 15-cm row spacing, 25-cm row spacing and 35-cm row spacing, respectively. The data are means ± standard error (SE; n = 3). Different small letters in each group indicate significant differences at *P*<0.05.

The response of LAI to row spacing was consistent during the two growing seasons (Fig 6). In both cultivars, the duration of green leaf area in R_1_ treatment was longer than that in other treatments since 10 DAA. At 30 DAA, on average, Jimai22 and Wennong6 in R_1_ exhibited a 22.3% and 30.5% increase, respectively, in LAI compared with conventional row spacing (R_2_). However, the LAI in the 5-cm row spacing treatment (R_0_) tended to be higher only in the period before 10 DAA, and it decreased dramatically thereafter. Meanwhile, LAI in R_3_ was always lower than other treatments. In addition, Wennong6 in R_1_ exhibited a higher LAI than Jimai22 during grain filling, which provided enough source leaves for grain filling.

### Light transmission ratio (LT)

**Fig 7:**
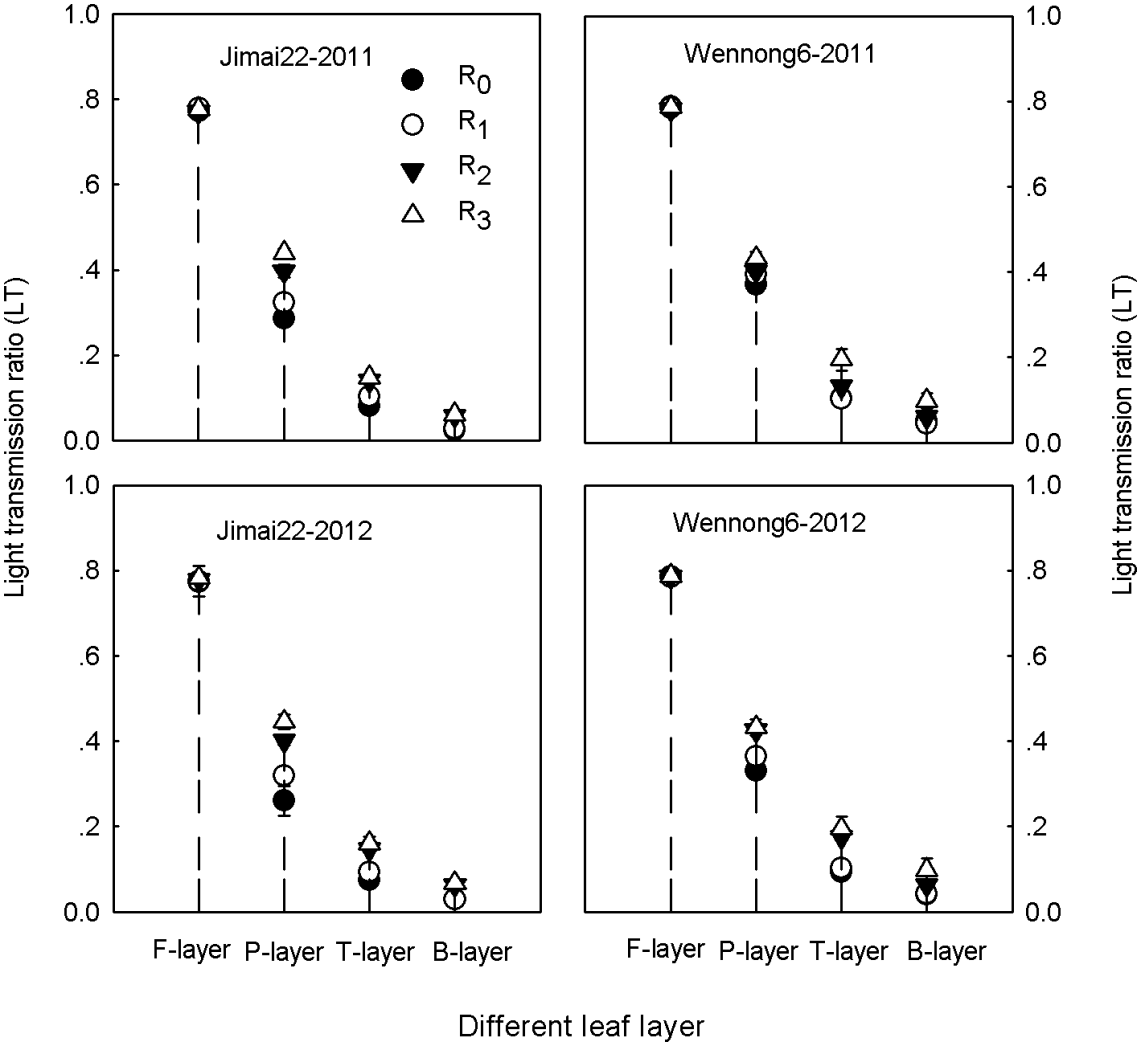
Effects of row spacing on light transmission ratio (LT) at mid-grain filling of two spike types of wheat under high planting density over a two-year period (2011 and 2012). R_0_, R_1_, R_2_ and R_3_ refer to 5-cm row spacing, 15-cm row spacing, 25-cm row spacing and 35-cm row spacing, respectively. F-layer, P-layer, T-layer and B-layer indicate flag leaf layer, penultimate leaf layer, third leaf layer and bottom leaf layer, respectively. Data are means ± standard error (SE; n = 3). Vertical bars indicate the SE.

The LT within the canopy was influenced by row spacing throughout the experiment (Fig 7). A significantly reduced LT was observed in the P-layer, T-layer and B-layer compared with that in the F-layer. During the whole grain filling stage, LT at different leaf layers increased as row spacing was widened. In addition, no significant differences were found in LT values at the F-layer, while LT at P-layer, T-layer, and B-layer in R_3_ treatment was significantly higher than that in R_1_ and R_0_, indicating that increased row spacing under high plant density improved the light condition within canopy (Fig 7).

### Grain yield, yield components and aboveground biomass at physiological maturity

Row spacing allocations had a significant effect on wheat yield over the two growing seasons (Table 1). The highest yield was observed for R_1_ during both experimental years. Wennong6 plants in R_1_ treatment exhibited a 21.0% and 19.1% increase in grain yield in 2011 and 2012, respectively, when compared to conventional row spacing (R_2_). The corresponding increase in Jimai22 plants was 11.3% and 11.4%, respectively, indicating that the large-spike cultivar is better adapted to high plant density under narrow row spacing. High yield values were obtained for R_1_ due to higher spikes per hectare. Moreover, aboveground biomass accumulation was influenced by row spacing, and 15-cm row spacing produced greater biomass than other treatments.

**Table 1:** Effects of row spacing on grain yield, yield components and aboveground biomass at physiological maturity under high planting density over a two-year period (2011 and 2012).

Yield structure	Treatment	Wennong6		Jimai22	
		2010–2011	2011–2012	2010–2011	2011–2012
	R_0_	482.76a	492.01a	734.19a	744.12a
Spikes (×10^4^•ha^-1^)	R_1_	460.38b	471.85a	718.18a	721.37a
	R_2_	424.87bc	430.16b	648.34b	662.41b
	R_3_	407.34c	409.94b	615.28b	614.82b
	R_0_	39.17d	38.51c	28.17d	27.48c
1000-kernelweight (g)	R_1_	42.53c	41.78c	30.35c	29.61c
	R_2_	43.27b	42.77b	32.07b	31.38b
	R_3_	44.76a	44.08a	33.94a	32.49a
	R_0_	5758.89d	5659.83d	5401.54c	5354.19c
Grain yield (Kg•ha^-1^)	R_1_	7394.67a	7209.38a	6503.92a	6453.09a
	R_2_	6109.65b	6055.82b	5841.87b	5791.82b
	R_3_	5958.92c	5879.29c	5507.94c	5419.21c
	R_0_	11835.12c	11078.83d	11300.29c	11131.37c
Aboveground biomass (Kg•ha^-1^)	R_1_	16432.63a	16384.95a	14781.64a	14666.11a
	R_2_	14917.25b	14418.62b	13909.21b	14200.52b
	R_3_	12034.72c	12248.52c	11217.80c	11104.94c

Note: R_0_, R_1_, R_2_ and R_3_ refer to 5-cm row spacing, 15-cm row spacing, 25-cm row spacing and 35-cm row spacing, respectively. The data represent the mean ± SE (n = 3). a-d Values followed by different letters within columns for the same cultivar are significantly different at the 0.05 probability level.

### Correlation analysis

**Fig 8:**
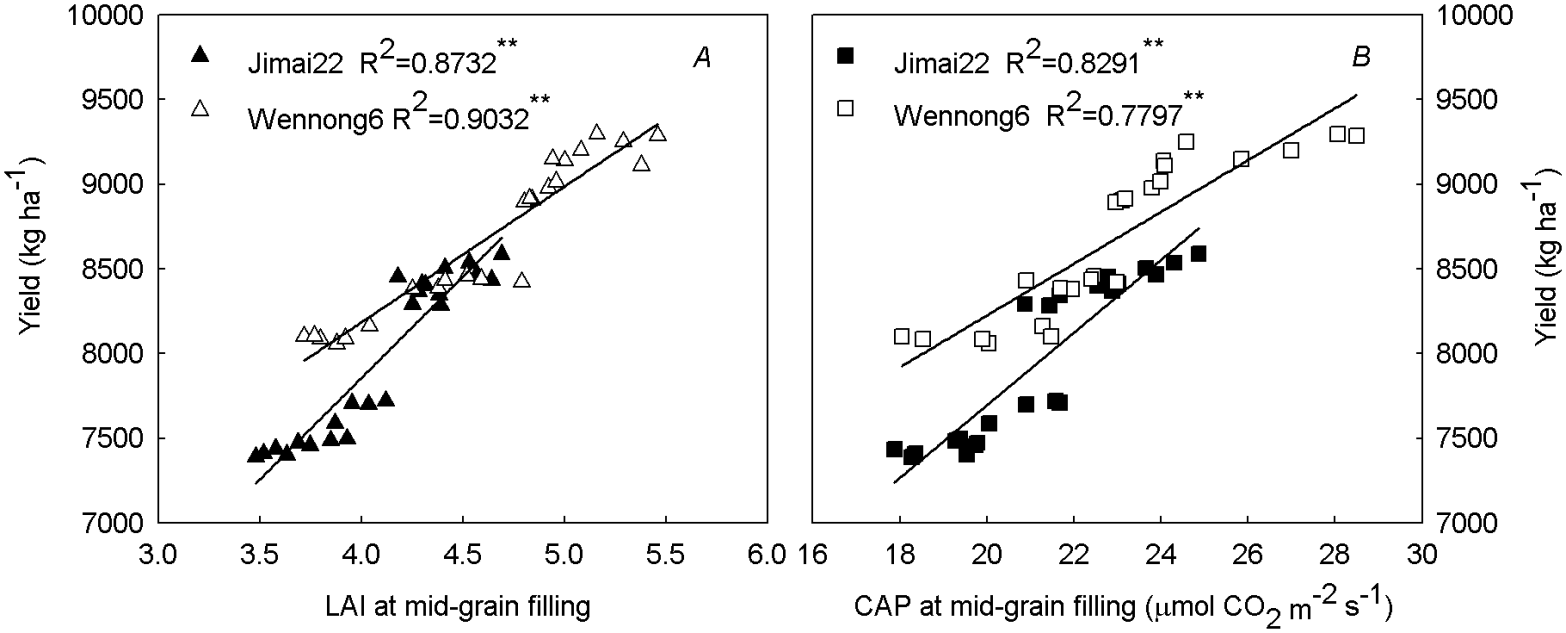
Relationships between LAI (*A*), CAP (*B*) at mid-grain filling stage and yield of two spike types of wheat under high plant density. Correlation coefficients (R) were calculated, and asterisks (**) represent the significance at the 0.01 probability level (n = 24).

A correlation analysis demonstrated that LAI and CAP values at mid-grain filling stage were both positively (*P* < 0.01) related to yield (Fig 8).

## Discussion

### Effect of row spacing on canopy structure of two spike-type wheat under high plant density

Plant distribution, which is partially determined by cultural practices, such as row spacing, affects canopy structure and yield [25–27]. Barbieri *et al*. [28] reported that reducing the distance between rows could increase radiation interception within the canopy and grain yield, which partially agrees with our results. In this study, compared with 25-cm conventional row spacing, reducing the distance between rows to 5 cm increased LAI only at anthesis, and then it declined dramatically. The reason why LAI in the S_0_ treatment markedly declined after anthesis may be related to the poor light conditions within canopy that accelerated leaf senescence (Fig 7). Since 10 DAA, higher LAI persisted for a longer period under 15-cm row spacing than other treatments (Fig 6), which allowed the plants to maintain a higher capacity of assimilate supply [29]. In contrast, increasing the distance between rows to 35 cm decreased LAI significantly during grain filling. Previous studies have also reported that increased row spacing improved light conditions within canopy [10]. At mid-grain filling stage, compared with 5-cm row spacing, increasing the distance between rows obtained higher LT values at the P-layer, T-layer, and B-layer (Fig 7), leading to higher P_N_ in penultimate and third leaves (Fig 4), which was beneficial for the improvement of individual photosynthetic capacity. However, excessive light leakage losses in R_3_ treatment were not favorable to improved irradiation energy utilization efficiency. According to the present results, we concluded that higher LAI in 15-cm row spacing enabled plants to make full use of light resources [30], which was likely due to an increased intra-row distance that delayed plant senescence rate [31–32]. Hence, under high plant density, 15-cm row spacing helped to form an effective canopy structure (longer duration of higher LAI and improved LT; Figs 6 and 7) during grain filling [33]. In addition, compared with Jimai22, Wennong6 had a more rational canopy architecture under 15-cm row spacing than Jimai22 (improved LT and higher LAI).

### Effect of row spacing on group and individual photosynthetic characteristics of two spike-type wheat under high plant density

CAP had a close relationship with canopy structure, which was influenced by row spacing, and ultimately affected grain yield [22, 34]. The CAP of traditional row spacing decreased with an increase in plant density, while it was enhanced under wide row spacing [30]. However, our results indicated that plants in 15-cm row spacing maintained a longer duration of CAP compared with the conventional row spacing during the most critical periods of grain setting, and the advantage of group photosynthesis was more obvious along with the grain filling process (Fig 3). In contrast, 35-cm row spacing decreased CAP significantly in spite of improved individual plant photosynthetic capacity (Fig 4), which was mainly attributed to the significantly lower LAI (Fig 6). According to our present results, we concluded that the reason why 15-cm row spacing maintained a longer duration of CAP was closely related to the efficient canopy structure (the longer duration of high LAI and enhanced LT) after anthesis. On the other hand, the higher *P*_N_ of penultimate and third leaves under 35-cm row spacing at the mid and late grain-filling stages did not compensate for the decline in LAI under high plant density (Fig 4) [33], resulting in a lower CAP. Moreover, this suggested that the higher tolerance of plants to high plant density under 15-cm row spacing was likely due to a more uniform spatial distribution and less in-row-plant-to-plant competition, resulting in a better coordination of CAP and individual plant photosynthetic capacity, ultimately achieving a higher grain yield.

Previous studies on CAP of different spike-type wheat cultivars have reached different conclusions. Xu and Zhao [35] found that large-spike wheat maintained greater CAP after anthesis. However, other studies found that CAP of small-spike wheat was higher [36]. In this study, the CAP of the two spike-type wheat was influenced by row spacing. However, the effects of row spacing on Wennong6 were more apparent than for Jimai22, and a greater CAP was observed in Wennong6 for 15-cm row spacing (Fig 3). This was in agreement with Guo *et al*. [37], indicating that large-spike type cultivar has higher canopy photosynthetic capacity by increasing plant density through optimum row spacing allocation (15 cm).

### Responses of yield and yield components to row spacing under high plant density

In this study, more biomass was produced at the 15-cm row spacing than other row spacing treatments, indicating better resource utilization in narrow rows than wider rows, which provided enough photosynthate for grain filling. Previous studies found that the yield of durum wheat (*T*.*aestivum* L.) was slightly improved at 30-cm row spacing under zero-tillage relative to that in l0- and 20-cm spacing [18]. In the present study, the greater grain yield was found at the 15-cm row spacing (Table 1). This result agreed with McLeod *et al*. [19], who reported that the number of spikes per square meter and grain yield decreased as row spacing increased. Fig 8 shows a positive correlation of yield with CAP and LAI. According to the present results, we concluded that the two cultivars when grown with a 15-cm row spacing maintained a higher duration of CAP and a higher LAI during grain filling as well as greater biomass accumulation, which provided more photosynthate for grain filling, leading to a significantly larger grain yield (Table 1). However, the yield increase extent of the two spike-type wheat under 15-cm row spacing was different. Compared with small spike-type wheat cultivar “Jimai22”, large spike-type wheat cultivar “Wennong6” had a more rational canopy architecture (improved LT and higher LAI) and CAP as well as better single-plant productivity (the heavier 1000-kernel weight), leading to 21.0% and 19.1% higher grain yield in 2011 and 2012, respectively.

## Conclusions

Over two years of experimentation, we found that reducing the distance between rows to 15 cm, when two spike-type wheat cultivars were planted under high density, optimized plant canopy architecture that maintained longer green leaf area duration, enhanced photosynthetic capacity of group and promoted greater biomass accumulation, resulting in an increased grain yield. Compared with 25-cm conventional row spacing, large-spike type wheat cultivar “Wennong6”in 15-cm row spacing obtained a 21.0% and 19.1% higher grain yield in 2011 and 2012, respectively, while for small-spike type wheat cultivar “Jimai22”it increased by 11.3 and 11.4%, respectively. The reasons for Wennong6 obtaining a higher grain yield were attributed to both a more rational canopy architecture (higher LT and LAI) and a higher CAP in 15-cm row spacing treatment, leading to a better coordination of group and individual leaf photosynthesis. This indicats that large-spike wheat has the potential to increase yields by increasing plant density through optimum row spacing.

## Supporting Information

S1 Dataset S1 Dataset contains data on climate data (Fig 1), canopy apparent photosynthesis (Fig 3), photosynthetic rate (Fig 4), F_v_/F_m_ and Φ_PSII_ (Fig 5), leaf area index (Fig 6), light transmission ratio (Fig 7), and correlation analysis (Fig 8). (XLSX)
